# Grey wolf optimized neural network with hybrid feature extraction for heart disease prediction

**DOI:** 10.1038/s41598-026-57798-3

**Published:** 2026-06-18

**Authors:** Sura Abdulateef Al-Shammari, Ahmad Sufril Azlan Mohamed, Ammar Abdulateef Hadi, Ahmed Abed Mohammed, Lamyaa Sabeeh Ashour, Omar J. Al-Karkhi, Dhafer G. Honi, Laszlo Szathmary

**Affiliations:** 1 Scientific Research Commission, Baghdad, Iraq; 2 School of Computer Science, University Sains Malaysia, Penang, 11800 Malaysia; 3 College of Computer Science and Information Technology, University of Al-Qadisiyah, Al Diwaniyah, Iraq; 4 College of Technical Engineering, Islamic University, Najaf, Iraq; 5 Department of Mathematics, College of Basic Education, University of Misan, Misan, Iraq; 6 Doctoral School of Informatics, University of Debrecen, Debrecen, Hungary; 7 Department of Computer Science, College of Education for Pure Sciences, University of Basrah, Basrah, 61004 Iraq; 8 Department of IT, University of Debrecen, Debrecen, H-4002 Hungary

**Keywords:** Heart disease prediction, Hybrid features extraction, SVM, CNN, ANN, GWO, Computational biology and bioinformatics, Diseases, Mathematics and computing

## Abstract

With an increasing mortality rate due to heart diseases, there is a critical need for early and reliable cardiovascular disease prediction. However, when it comes to health records data, traditional approaches have primarily utilized a single prediction model. This study proposes an advanced approach that leverages Support Vector Machine (SVM) in independent parallel streams with Convolutional Neural Networks (CNN) for extracting features, combined with an ANN enhanced via Grey Wolf Optimization (GWO) for predictive classification. First, the input data is passed into the SVM and CNN simultaneously to extract relevant and important features by each method. Both extractors receive identically preprocessed inputs simultaneously; their outputs are concatenated post-extraction. The SVM extracts probability-based discriminative features reflecting class separation confidence, while CNN extracts hierarchical convolutional features capturing local patterns and non-linear attribute interactions. Then, the attributes extracted by SVM and the attributes extracted by CNN are concatenated to get a comprehensive set of features. The concatenation enriches the feature space with diverse representational characteristics: SVM contributions provide regularized decision confidence scores, while CNN contributions offer multi-scale pattern descriptors. This heterogeneous feature integration enables the ANN to learn more robust classification boundaries by accessing both discriminative and descriptive feature domains simultaneously. Second, the concatenated features are sent to the ANN for prediction. The GWO algorithm is used to configure ANN parameters like channel size, drop-out rate, and learning rate. The fitness function maximizes stratified 5-fold cross-validation accuracy. The experiments were conducted on the Heart Disease dataset collection, collected from the UCI Machine Learning Repository (Cleveland, Hungarian, Switzerland, and Long Beach), which includes 14 variables for heart disease prediction. All results represent mean ± standard deviation from nested cross-validation. The results demonstrate that the proposed SVM–CNN+ANN-GWO model achieves a high classification accuracy of 91.80% ± 1.2% with the Cleveland, 91.53% ± 1.5% with Hungarian, 96.08% ± 0.8% with Switzerland, and 87.69% ± 2.1% with VA Long Beach databases, respectively. Statistical significance was confirmed via paired Wilcoxon tests (*p < 0.05*). It outperforms existing baseline approaches and other recent approaches from previous studies based on predictive accuracy. It achieves promising performance in heart disease prediction across all four databases and hence generalizes well on different datasets.

## Introduction

Cardiovascular disease (CVD) is defined as condition that impacts the cardiovascular system. The mechanisms that cause certain illnesses are distinct from one another. Dietary risk factors are responsible for around 53% of cardiovascular disease deaths^1^. Atherosclerosis is a component such as disorders affecting the coronary arteries, stroke, and diseases of the peripheral arteries. Approximately 90% of cardiovascular diseases might be avoidable^2^. Eating well, exercising regularly, not smoking, and limiting alcohol use are all ways to improve risk factors and prevent them. In 2015, CVD accounted for 17.9 million deaths (32.1%), up from 12.3 million (25.8%) in 1990^3^. At certain ages, cardiovascular disease (CVD) causes of death are more common and have increased in most developing nations, whereas rates have declined in most developed countries since the 1970s^4^.

Artificial Intelligence (AI) represents a part of technical science that feeds computers with the ability or machines with human-like intelligence^5^. Hence, the most popular field of applied AI is machine learning, which refers to the process involved in training models or algorithms to perform desired tasks that are common in various fields such as satellite^6,7^, agricultural^8,9^, healthcare^10,11^, and more. Machine learning is extensively used in the medicine domain and healthcare industry as a smart tool to analyse data and assist doctors as well as healthcare providers in making better decisions related to treatment and patient care. Hence, there is an acute need for leveraging technology to predict heart conditions quickly, efficiently, and accurately^12^. As of today, a vast set of data having relationships with heart disease patients is available, and it can be used to discover patterns, relationships, and other useful information for heart disease diagnosis. Despite significant advancements in predicting heart disease, conventional approaches often face challenges in accurately identifying subtle cardiac anomalies in the early stages^13^. Standalone ML or DL models struggle to fully capture the complex patterns within structured medical data, and manually tuning model hyperparameters is inefficient and can compromise model generalizability^14,15^. Moreover, predictive systems of heart diseases highly rely on the reliability of patient records and the robustness of the assessment strategy. Openly prepared benchmark datasets allow one to fairly compare various predictive models and use them as an objective point of reference to test new methods. Nevertheless, differences in data distribution, patient characteristics, and sample sizes across datasets usually disrupt consistent performance and constrain the generalisation of most currently available models. As such, predictive frameworks should be tested on diverse datasets using standard performance measures to demonstrate their consistency and practical use. An in-depth experimental investigation of the datasets can offer deeper insight into the effectiveness of feature expression, categorisation, and optimisation methods, and can also underscore the usefulness of intelligent decision-support systems for identifying early heart disease.

To resolve these challenges, this study proposes to improve the early detection methods for CVD using machine learning techniques. The proposed technique seeks to harness the combination of hybrid feature extraction ML, DL approaches and hyperparameter optimization of the Artificial Neural Network (ANN) diagnostic model with the GWO algorithm for more accurate and efficient diagnostic procedures. It leverages SVM and CNNs to obtain significant features from the data, operating in parallel computational streams with aligned outputs, and passes the features to a neural network used for disease prediction. Standalone ML or DL models struggle to fully capture the complex patterns within structured medical data. SVM excels at margin-based discrimination and outlier handling, while CNN automatically learns hierarchical spatial representations. Their combination cultivates complementary feature perspectives, mitigating individual limitations and enhancing discriminative capacity for clinical prediction tasks. The GWO method was used to identify the best architecture for the ANN.

The contributions of this paper are:

1. Proposing an improved heart disease detection system that utilizes a hybrid feature extraction (SVM-1DCNN) method and performs prediction by ANN. The parallel extraction mechanism and explicit feature dimension documentation ensure reproducibility.
2. Utilizing the GWO algorithm to improve the proposed ANN’s performance by fine-tuning parameters to maximize accuracy for early and precise heart disease prediction. Full GWO configuration and search space bounds are provided for replication.

The paper is organized as follows: Section 2 reviews related studies on feature extraction and detection models for heart disease. Section 3 details the proposed methodology, including the dataset, preprocessing steps, the hybrid SVM-1DCNN feature extraction, the ANN prediction model, and the GWO-based hyperparameter optimization. Section 4 shows and analyzes the results, comparing the performance of the base model with the GWO-optimized model and against previous studies. Finally, Section 5 provides concluding remarks and proposes potential areas for further investigation.

## Related studies

### Feature extraction techniques for heart disease prediction

Satpura et al.^16^ utilized SVM to detect heart disease on the heart disease dataset from the UCI database. They used the PSO optimizer to enhance the SVM parameters and K-fold cross-validation to boost its predictive performance even further. There were no preprocessing steps taken. Initially, they obtained a classification accuracy of 81.85% along with an AUC score of 0.823. When they used the PSO algorithm to adjust the model parameters of SVM, the accuracy increased to 84.81%, and the AUC value increased to 0.898. As a result, the study found that using an optimization algorithm can enhance the overall performance of the performance of the ML models for predicting heart disease. Ricciardi et al.^17^ predicted the prevalence of coronary artery disease among patients through a custom dataset that includes data from 10,265 patients. The dataset contains information related to traditional cardiovascular risk factors, closely to the cardiovascular disease dataset from the UCI repository. They employed PCA for extract of feature and then LDA for prediction. Features were extracted using PCA, and the performance of the prediction model with PCA and without PCA was compared. Without PCA, they achieved an accuracy of 84.5%, and with PCA, they got 86%. Hence, feature extraction was successful in improving the performance of the predictive model. Moreover, feature extraction leads to better management of large datasets and faster training by providing new features that are as good as the original ones. Reddy et al.^18^ utilize the Cleveland dataset to predict heart disease by applying PCA-based feature extraction and sending it to several models for further processing and prediction. The performance of individual models and models trained on PCA extracted features was compared. The models performed better when PCA was used, based on the results. The best accuracy obtained by the Logistic Regression model was 85.8%. This model was trained on 9 features that were extracted using the PCA method. The study further strengthened the function of feature extraction processes in improving predictive performance of the models and emphasized the significance of combining methods to achieve better performance. Kumari^19^ advised using Bi-LSTM and Random Forest together to predict the occurrence of cardiac disease. Here, first the data was fed into the Random Forest model for feature extraction, and then those were sent to Bi-LSTM for subsequent feature extraction and prediction. The method achieved 90% accuracy according to the Cleveland heart disease dataset from UCI (training data) and demonstrated that combined approaches (ML+DL) are suitable for providing better diagnostic options for heart disease in patients. When compared to other existing methods, it performed significantly better with significantly higher accuracy, emphasizing the importance of hybridization even more. Shankar et al.^20^ determined whether the person is likely to develop cardiovascular disease using a CNN model. Their prediction model was trained and evaluated over real-life hospital data in text format. The convolution layers were utilized to extract features, and the generated features were sent to the last layers for prediction. The system utilized a CNN model to perform both tasks and attained a maximum accuracy of 88% in multiple trials. Deep neural network performance methods outperformed those of Naïve Bayes and KNN algorithms, indicating that they are more successful at extracting features from medical data and making predictions based on them. Vani^21^ predicted CVD using a dynamic LSTM model, in which the top layers extracted features and the bottom layers predicted. They used LSTM’s adaptive threshold structure to learn the temporal characteristics of the dataset and use it to predict CVD. However, the main objective of their work was to improve the input of the internal forgetting gate in order to overcome prediction obstacles, resulting in a dynamic LSTM model. The suggested model obtained an accuracy of 89.6% using the surrogate dataset, demonstrating that the dynamic LSTM model outperformed the conventional LSTM model. Hybridization, or the combination of two or more methods, can lead to further advancement. Almulihi et al.^22^ implemented two different heterogeneous hybrid: CNN-LSTM and CNN-GRU models for the prediction of heart diseases. Using the stratified sampling method, to split the dataset between the two phases, an 80:20 data division was applied for model development and testing. In this case, feature extraction was carried out using the CNN model, and LSTM & GRU were used respectively for prediction. The architecture is a follows: a convolution layer, a maximum pooling layer, a GRU/LSTM layer, a flatten layer, a dense layer, and an output layer. The models were fitted on the UCI Cleveland dataset and were optimized utilizing the Bayesian Optimiser. The accuracy produced by the hybrid CNN-LSTM model was 89.76%, while the CNN-GRU model reached 88.29% accuracy. The efficacy of CNN for feature extraction and overall improvement of predictive performance was demonstrated. It is proposed to test the hybrid approach on other datasets as future work.

Recent advances in optimization-based prediction include GA-PSO-SVM models for compound fault diagnosis^44^ and Schrödinger equation-based adaptive dropout networks for feature enhancement^45^. Network enhancement methods for biomedical prediction^46^ and hybrid module integration approaches^47^ demonstrate the efficacy of combining heterogeneous techniques in clinical prediction. Feature selection methodologies for heart failure prediction using correlation-based approaches^48^ and hybrid CNN integration for medical image analysis^49–51^ demonstrate the broader applicability of fused feature strategies in clinical prediction.

### Prediction models for heart disease

Anderies et al.^23^ compared the performance of several machine learning algorithms on the UCI Cleveland dataset. The models developed were SVM, Naive Bayes, Logistic Regression, Neural Network, Decision Tree, and KNN. During the evaluation, the accuracy of SVM was 85%, followed by LR and NB, both with an accuracy of 83.33% each. The NN model obtained an accuracy of 80%, whereas the KNN obtained 78% and the Decision Tree reached 70%. Therefore, the SVM emerged as the best possible model for predicting binary outcomes of this data. Ramesh et al.^24^ compared the effects of ML models with statistical techniques for the case of heart disease prediction. They built various ML models and evaluated their performance on the UCI Cleveland heart disease dataset. The accuracy attained by Decision Tree was 78.98%, Naive Bayes was 86.78%, Random Forest was 87.9%, SVM was 89.1%, and Logistic Regression was 87.1%. The highest performance was obtained by the SVM model, and it outperformed the statistical methods, indicating that ML-based methods are most suitable for heart disease prediction. Ananey-Obiri and Sarku^25^ built and compared multiple ML models for detecting the presence of heart disease. The models that were selected were DTs, NB, and LR, and they obtained accuracies of 79.31%, 76%, and 82.75%, respectively. In the experiment, Logistic Regression outperformed the other models, and the research showed that there has to be a workaround for features in the data so that to improve the predictive performance. Mary et al.^26^ investigated the assessment of different prediction models for heart disease risk prediction. Their goal was to increase prediction accuracy and minimize errors rates, so they ran 10-fold cross-validation on each model before making predictions. SVM had the greatest accuracy of 83.49%, followed by Naive Bayes at 82.80% and Logistic Regression at 82.10%. The study results show that cross-validation helped to improve performance, and as future work hybridisation approach can be used. Rani et al.^27^ introduced a clinical decision-support framework for predicting heart disease. The Random Forest model obtained the best accuracy of 86.6% when it was assessed on the Cleveland heart disease dataset. The accuracy of Logistic Regression was 85.07%, Adaboost achieved an accuracy of 86.59%, SVM obtained an accuracy of 84.46%, and Naive Bayes had an accuracy of 83.55%. Islam et al.^28^ observed the Performing of Logistic Regression, SVM, Decision Tree, and Naive Bayes algorithm on the UCI Cleveland dataset. The respective accuracies of Logistic Regression, SVM, Decision Tree, and Naive Bayes are 82.9%, 86.1%, 85%, and 75.8%. They found that SVM outperformed the other models for the UCI dataset. Amen et al.^29^ investigated the ML techniques of SVM, Logistic regression, random forest, gradient boosting, and extra trees. The resulting accuracy of SVM was 80%, LR was 82%, RF was 77%, GB was 74%, and Extra Trees was 79%. LR and SVM were the two best algorithms for the binary prediction task. Javid et al.^30^ implemented LSTM and GRU models individually to predict cardiovascular diseases. The UCI Model training and evaluation were conducted using the heart disease dataset, achieving accuracies of 81.31% for LSTM and 81.46% for GRU, respectively. However, when these were tied into an ensemble with other machine learning models (SVM, KNN, & RF), the accuracy improved to 85.71%. To conclude, the research showed that combination approaches, such as ensemble, surpass the prediction accuracy of every model. El-Shafiey et al.^31^ developed a hybrid DL-based model consisting of Bi-direction LSTM and one dimensional CNN for heart illness detection. The hyperparameters are optimized using the Bayesian optimizer, and to control overfitting, they used dropout layers in the CNN architecture. Training of the models is performed across two datasets, Cleveland and Statlog. The Cleveland dataset recorded an accuracy of 89.51%, whereas the Statlog dataset yielded an accuracy of 82.72%, respectively. Harkulkar et al.^32^ detected heart disease using the 1D-Convolutional Neural Networks and the UCI Cleveland dataset. The experiment recorded an accuracy of 75.2% and showed that DL methods are suitable for the analysis of heart illness. Khan Mamun and Elfouly^33^ used 1D-CNN and ANN algorithms for the prediction of CVD. The National Health and Nutrition Examination Survey by the CDC database was used for training and evaluation of the model. The 1D-CNN model achieved an accuracy of 80.03%, surpassing the accuracy of the ANN model, which was 75.74%. Biswas et al.^34^ conducted experiments on the UCI heart disease dataset (total of 1190 samples and 14 attributes) with CNN, LSTM, and ANN algorithms for early prediction of heart illness. The results achieved with the ratio of 90:10 include accuracies of 87%, 86%, and 83% for CNN, LSTM, and ANN models, respectively. Cherian et al.^35^ detected heart illness utilizing the UCI heart illness data, feature selection methods, and ANN algorithms. The primary objective of this paper was to achieve as optimal accuracy as possible, and the ANN model with optimization recorded an accuracy of 87.09%. Novichasari and Wibisono^36^ presented the utilization of PSO to enhance the efficiency of detection models like ANN. When trained on the heart illness dataset obtained from the UCI repository, the ANN model with PSO optimization achieved an accuracy of 85.5%. It is important to note that without PSO, the ANN had only 77% accuracy. As a result, the study emphasizes and strengthens the use of a specific metaheuristic algorithm for improving the accuracy of deep learning approaches. In subsequent studies, the work could be expanded with additional special algorithms.

## Methodology

**Fig. 1:**
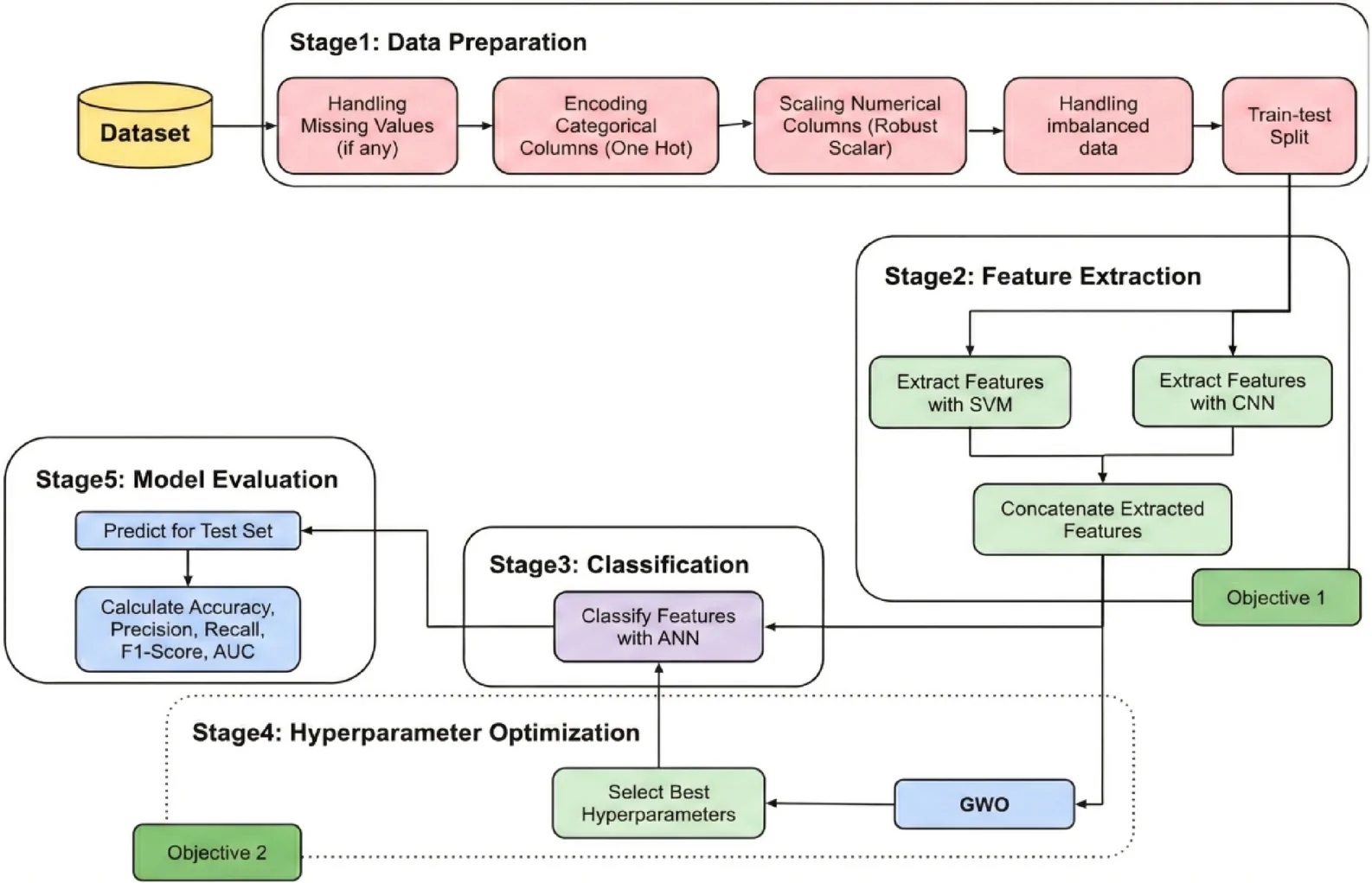
Stages of the proposed approach.

This study proposes an enhanced technique for heart disease diagnosis due to the limitations of individual models applied to predict cardiovascular disease. The proposed method uses a hybrid feature extraction approach and Grey Wolf Optimization (GWO) algorithm-optimized Artificial Neural Network (ANN) for the prognosis of cardiovascular disease. The aim is to analyse how a hybrid model may extract relevant attributes from clinical records and how the performance is enhanced with the help of the GWO algorithm-based fine-tuning. Figure 1 demonstrates the stages of the study.

### Dataset description

This study utilises the Heart Disease dataset from the UCI repository, which comprises four datasets: Cleveland, Hungarian, Switzerland, and VA Long Beach. This diversity in data sources enhances the credibility, generalizability, and robustness of research findings. The database captures variations in patient populations, healthcare systems, and geographical locations. Each dataset has 76 columns that are related to factors of heart health, but almost all researchers have focused on 14 important features Janosi et al. in 1988^37^. The target variable represents whether a person is healthy or has CVD (0 indicates no heart disease). They were used by Wadhawan and Maini in 2021^38^. Table 1 displays the shape and target information associated with each of the databases.

**Table 1:** UCI dataset characteristics.

Database	No. of features	No. of observations	No. of target categories
Cleveland	14	303	2
Hungarian	14	294	2
Switzerland	14	123	2
VA Long Beach	14	200	2

- Cleveland dataset: The Cleveland dataset comprises 303 instances related to heart disease patients, which were collected in the Cleveland Clinic Foundation by Robert Detrano, M.D., Ph.D. The dataset includes 139 patients with heart disease issues, while 164 patients did not exhibit any problems. Most researchers choose the Cleveland dataset as their benchmark because it contains more samples and is known for its cleanliness. Cleveland is a dataset that is almost balanced in terms of data and, therefore, does not require any preprocessing steps for handling imbalanced data.
- Hungarian dataset: The Hungarian dataset contains data from 294 patients. There are 106 instances with positive heart disease and 188 instances with negative heart disease. The database was obtained from the Cardiology Institute in Budapest, Hungary, by Andras Janosi, M.D. The database contains numerous missing values. Additionally, it is an imbalanced dataset and requires management of these issues during preprocessing.
- Switzerland dataset: The Switzerland dataset consisted of 115 cases of heart illness and 8 individuals without heart disease, totaling 123 instances. Drs. William Steinbrunn and Matthias Pfisterer of Switzerland’s University Hospitals in Zurich and Basel, respectively, gathered this data.
- VA Long Beach dataset: Gathered at the V.A. Medical Centre in Long Beach, by Robert Detrano, M.D., Ph.D. There are 149 cases with positive heart disease symptoms and 51 cases with negative heart disease symptoms, making a total of 200 instances. The imbalanced problems of this database, which displays the null value percentage present in every column of this dataset, should be addressed before it is sent for training. Additionally, it should be noted that during preprocessing, the majority of the columns have absent values, and some of them have a high percentage of null values.

### Preprocessing

**Fig. 2:**
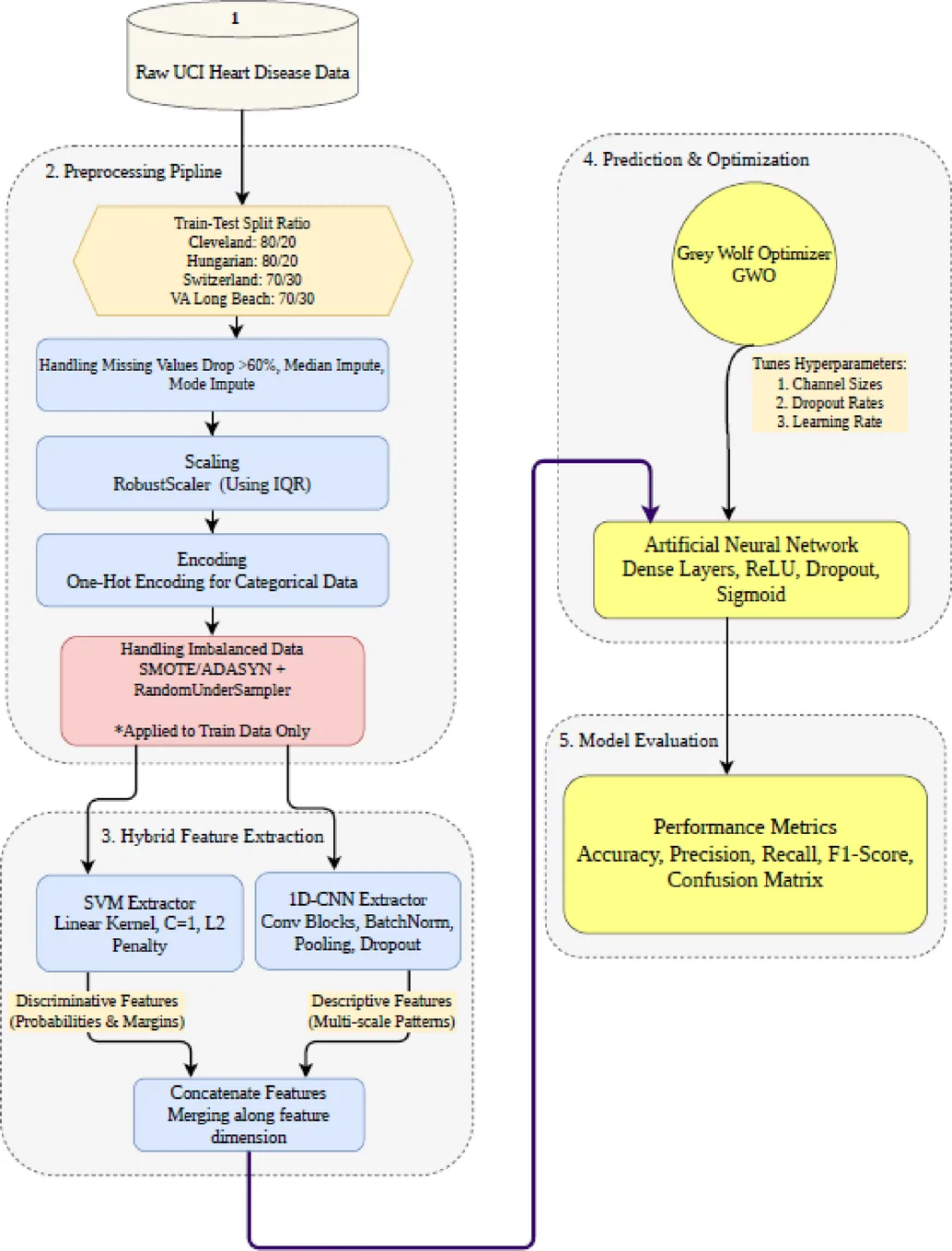
Visualizing split-aware preprocessing pipeline.

The most important step in developing a novel model is preprocessing. In prediction problems, the direct impact on the result and the reduction of bias and overfitting towards a specific class are achieved by having clean data through the handling of missing and skewed values. Additionally, the scaling of numerical data and the encoding of categorical data aim to enable models to recognize patterns and obtain valuable information from the data, resulting in the creation of a high-performing model quickly and accurately. In this work, various steps for data preparation have been adopted, including missing value treatment, categorical data encoding, normalization, and synthetic data generation. The preprocessing pipeline follows strict split-aware protocol to prevent data leakage: (1) train-test split performed first; (2) missing value imputation fitted on training data only; (3) RobustScaler statistics computed on training set; (4) categorical encoding derived from training categories; (5) resampling (SMOTE/ADASYN + RandomUnderSampler) applied to training data exclusively; (6) feature extractors trained on processed training data and transformed to test data as shown in Figure 2.

#### Handling missing values

In each dataset, there are various missing values and diverse types of data, which require different management methods. Firstly, if the rate of missing values in a column exceeds 60%, that column is discarded and excluded from the training process. Secondly, in numerical columns, the median value of that column is used to fill in the null values. Finally, for categorical data, missing values are filled with the most frequent value of the column. A unified framework was applied across all datasets: missing value threshold (*>60%* drop), numerical imputation (median), categorical imputation (mode), and RobustScaler normalization.

1. In Cleveland, the ‘ca’ column contains 87.4% missing values. Unlike other datasets where this column exceeds 90% missing and is dropped, Cleveland retains sufficient non-null entries (12.6%) for mode imputation. The most frequent value (0) was used, preserving this clinically relevant predictor.
2. In the Hungarian dataset, the number of missing values on “slope”, “ca”, “thal” columns are more than 60%, slope (65%), ca (99%), and thal (90%); thus, these columns were dropped from the dataset. Following that, the remaining null values are imputed: numerical columns use median, categorical columns use mode.
3. In the Switzerland dataset, the ’ca’ column has been dropped (because of 95% missing values), while other columns having missing values have been treated appropriately by filling nulls of numerical columns with the median of the column and filling nulls of categorical columns with the most frequent value of the column. The missing fbs (60.9%), slope (13.8%), and thal (42.2%) were filled with a fixed value of 4. This fixed value represents a clinically valid category for these variables.
4. The ‘ca’ as well as ‘thal’ columns in VA Long Beach, have more than 80% missing values with 99% and 83% respectively that are dropped. The missing ‘slope’ column is filled with 0 fixed value. Dropping ’ca’ and ’thal’ eliminates established clinical predictors, limiting real-world applicability. Future work will employ iterative imputation to retain these variables.

#### Scaling numerical columns

The distance between data points is crucial in some algorithms. Scaling, for example, is required in SVM to manage the high variance of numerical columns. Deep learning models are extremely sensitive to changes in magnitude of variable units. As a result, scaling has an impact on neural network performance. Furthermore, higher variable values increase training time and slow down the optimization process. Therefore, it is crucial to scale the variables before training the deep learning models. It will help ensure that the models train faster and error converges more quickly towards the minima^38^. Scaling is used in this work to transform the numerical features “age”, “trestbps”, “chol”, “thalach”, “oldpeak”, and “ca”, and bring them on a similar scale. The Robust Scaler was used in the implementation. Robust Scaler uses the Quantile Range and Interquartile Range (IQR) methods during scaling. The IQR selects the values between the first quartile (25%) and the third quartile (75%) with the following formula:

1 $$\begin{aligned} \textrm{IQR} = Q_3 - Q_1 \end{aligned}$$

The equation for this is denoted as:

2 $$\begin{aligned} X_{\text {new}} = \frac{X - X_{\text {median}}}{\textrm{IQR}} \end{aligned}$$

#### Encoding categorical columns

The Heart Disease datasets contain categorical variables. A categorical variable can take one of the fixed, specific, and limited numbers of values and usually defines a qualitative property. They represent information based on their names or labels and are most of the time in text format, like A, B, or C. However, in the cardiovascular disease datasets, the categorical variables are already in numerical format, such as 1, 2, or 3. In One Hot Encoding, for every distinct value within the categorical feature, a separate binary column is generated. If the value appears in an observation, the related column is set to 1, and everyone else’s columns are set to 0.

#### Handling imbalanced data

In this study, the best method between SMOTE and ADASYN as an over-sampler and RandomUnderSampler as an under-sampler is used. Firstly, the over-sampling is applied on minority classes with a specific sampling strategy rate, and then Random undersampling with a “previous strategy rate + 0.1” sampling strategy is performed to select all minority samples and samples from the majority class.

1. The Cleveland dataset is almost balanced; therefore, handling imbalanced data is not performed for it.
2. The Hungarian dataset has a small imbalanced issue. The various techniques were tried on this dataset, and the selected method achieved the highest performance. Thus, the training set of the database is balanced by using a combination of ADASYN with a 0.7 sampling strategy for oversampling and RandomUnderSampler with a 0.9 sampling strategy for undersampling.
3. The imbalance in the Switzerland data was handled using SMOTE-based oversampling through a 0.6 sampling strategy and RandomUnderSampler-based undersampling with a 0.7 sampling strategy.
4. Since the VA Long Beach database is imbalanced, same as Switzerland, it is managed by applying SMOTE and RandomUnderSampler.

**Fig. 3:**
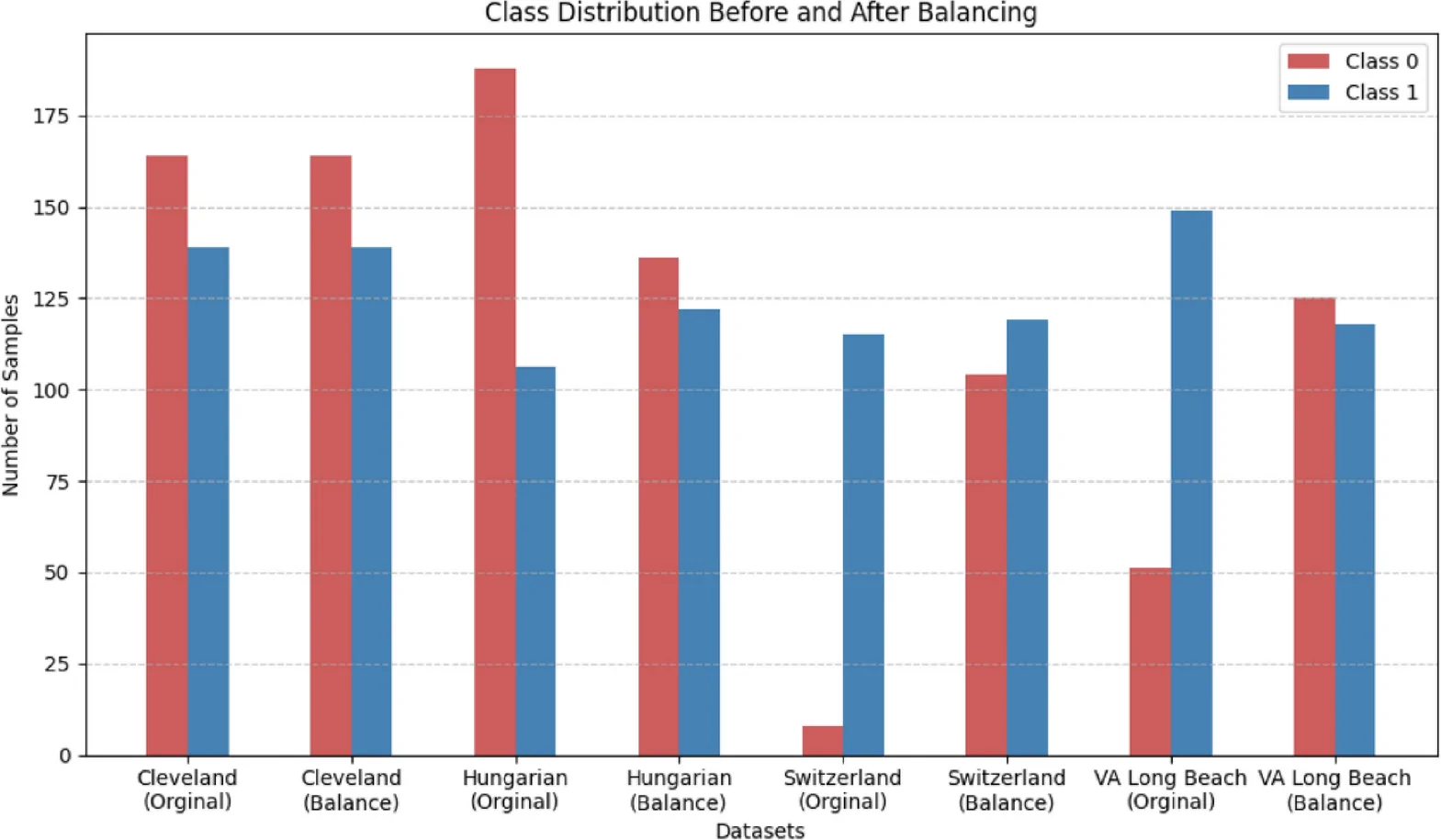
Datasets before and after balancing.

The distribution of the target variable in both respective datasets selected for this work is visualized in Figure 3. After balancing the training data to achieve nearly equal representations of both classes, the distribution of the target is changed. It is visualized.

### Hybrid feature extraction (1DCNN-SVM)

Feature extraction is the major process for most AI problems, including supervised and unsupervised problems such as regression and classification. The selection of appropriate features from the input data, which have the greatest impact on detecting the final goal, helps to reduce the time and cost complexity of the models. This has consistently attracted interest from numerous researchers. The effectiveness and importance of features for achieving more information while training are vital for having accurate models^39^. Sometimes the data hurt each other, and using all of them may result in decreasing performance of the model, while detecting and dropping these features is more beneficial, and it is the duty of the feature extraction part to find them^40^. The Convolutional Neural Networks (CNN) and Support Vector Machines (SVM), which are DL and ML techniques, respectively, are applied to extract features. The use of these two models helps to cultivate features from a diverse point of view and covers the weaknesses of each method, while also combining their power. The prediction algorithm can detect the presence or absence of disease more precisely in this manner. Feature extraction operates in parallel computational streams: SVM and CNN processes execute independently on identically preprocessed inputs. Both extractors yield two-dimensional outputs (samples *×* features) aligned by patient index. The concatenation merges along the feature dimension (axis=1), preserving sample alignment while expanding the feature vector. The explanation of the feature extraction models is provided in the subsequent subsections.

#### SVM for feature extraction

Here, SVM is also chosen because of its effectiveness in handling outliers, which are largely present in the dataset selected for this paper^41^. In this work, the SVM is used to extract features from the medical records data. SVM feature extraction employs the calibrated probability outputs (Platt scaling via 5-fold cross-validation) as transformed features. Specifically, for each sample, the probability estimate *P(y=1|x)* from the linear SVM decision function serves as a discriminative feature dimension. Additionally, the signed distance to the separating hyperplane provides geometric margin information. The process of feature extraction works as follows. A pipeline is defined for cultivating patterns and important characteristics among data. It contains SVM and is considered to be used for feature extraction, with its kernel set to linear. The SVM uses an L2 penalty to raise the power of regularization; thus, the parameter C = 1 is selected. Probability is the other parameter that is useful for enabling the probability estimator of SVM when the plan is to extract features. This active 5-fold cross-validation internally for it and returns the prediction probability as an output of the best-fitted model. In addition, the Class weight is set to balance. This gives inverse weight to the class with higher samples and helps to balance data during cross-validation. Next, the pipeline is fitted on the training data, and the important features are derived by applying transformations to the training data. Similarly, the same fitted transformer is used on test data to identify the same features while testing the final model. After fitting the SVM model on training data, a total of 7 features were extracted for Cleveland, 6 features were extracted for Hungarian, and 9 features for each of the Switzerland and the VA Long Beach datasets.

#### CNN for feature extraction

The CNN is constructed from one or more convolution and pooling layers that are used for finding patterns in data and extracting those unique and special features^33^. It convolves through data with the assistance of its kernel, which is a weight matrix, and extracts features. The kernel can have various sizes, and it is crucial to select an appropriate kernel size to prevent overfitting or underfitting^32^. Features are extracted from the flatten layer following the final pooling operation. The 1D-CNN processes tabular inputs reshaped as (features, 1) sequences, treating each attribute as a positional element. This representation enables convolutional kernels to detect local attribute correlations analogous to sequence patterns. The 1D-CNN model uses an assortment of convolutional, batch normalization, pooling, and dropout layers as convolutional blocks (Conv Block). Additionally, a flattening layer is employed to minimize the dimension of the dataset. The 1D-CNN model is constituted by a combination of several Conv Blocks sequentially. In this work, two Conv Blocks are used for CNN feature extraction. Each block contains a 1D conv layer with different parameters for each dataset. Also, a Batch Normalization layer that normalizes the outputs of the convolutional layer and helps to reduce the computational complexity of the model. Then, a pooling layer with a window size of 2 is applied to downsample the feature maps by dividing them into small regions and computing either the maximum or the average value within each region; this layer aims to decrease the dimension of data and transfer important attributes to the next layer, and its type is differentiated based on datasets. There is a Dropout layer in each Conv Block for omitting some neurons randomly based on the defined rate. Dropout helps the model to see different data and increases the regularization of the model.

**Fig. 4:**
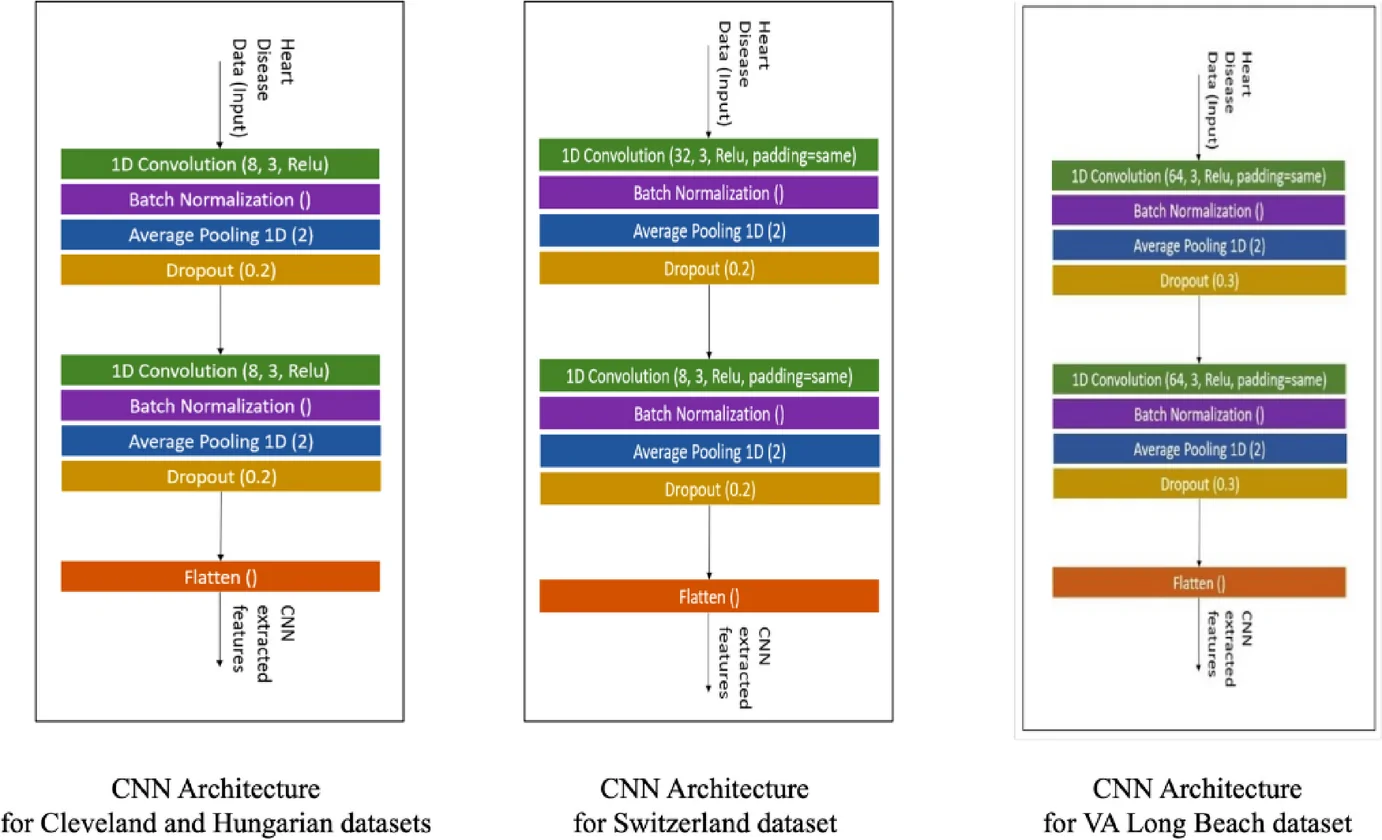
Architecture of CNN for each dataset.

For the Cleveland and Hungarian datasets, the CNN model has two CNN blocks, each of which contains: A 1D-CNN layer with a 32 filter size, kernel size 3, and ReLU activation function. A Batch Normalisation and an average pooling using a pooling size of 2. Following these layers, a Dropout layer with a drop rate of 0.2 is used to create diversity in data and control overfitting. After training the CNN, the 96 features were extracted by the 1DCNN model. The CNN model of the Switzerland database has two CNN blocks, each of 1D CNN has 32 filter sizes, kernel sizes 3, and with the Relu activation function. The padding of this layer is set to ‘same’ to increase the number of features because this dataset is highly imbalanced. The other layers of Conv blocks are a Batch Normalisation, Average pooling with pool size 2, and a dropout layer with 0.2 drop rate. It extracts a total of 160 features from this dataset. For VA Long Beach, the CNN model has 2 blocks. Each block consisted of a 1DCNN layer using 64 convolutional filters, kernel size 3, and rectified linear unit (ReLU) activation, then a Batch Normalization layer. Then a MaxPooling1D layer with pool size 2 and a Dropout layer with 0.3 drop rate. Aside from those two Conv blocks, an overall average pooling is added to this architecture to reduce the dimension of final features, and at the end, 64 features are extracted. The CNN model architecture for each dataset as illustrated in Figure 4.

#### Concatenate extracted features (1DCNN-SVM)

When different features link together and create a series of data, the concatenation happens. This joining can be done based on any dimension if the data has more than one dimension. Here, the features cultivated by SVM and CNN have two dimensions: the first one is samples per patient, and the second one is newly extracted features. As the plan is to add more information to the prediction algorithm while classifying each patient, thus the merge of features is done on the second one, this keeps the number of rows the same and increases the number of columns. The concatenation enriches the feature space with diverse representational characteristics: SVM contributions provide regularized decision confidence scores, while CNN contributions offer multi-scale pattern descriptors. This heterogeneous feature integration enables the ANN to learn more robust classification boundaries by accessing both discriminative and descriptive feature domains simultaneously.

**Table 2:** Concatenated feature dimensions.

Dataset	SVM features	CNN features	Total concatenated	Train/test split
Cleveland	7	96	103	80/20
Hungarian	6	96	102	80/20
Switzerland	9	160	169	70/30
VA Long Beach	9	64	73	70/30

### ANN algorithm for heart disease prediction

The ANN architecture of this work gets the features extracted by SVM and CNN as its inputs, which are concatenated together. The ANN model architecture will have several Dense layers that are followed by the Dropout layer and organized sequentially to achieve the desired goal. The ANN architectures were determined through empirical architecture search. Cleveland and Hungarian datasets employ six dense layers due to their larger sample sizes and feature complexity. Switzerland and VA Long Beach use two dense layers reflecting their smaller sample sizes to prevent overfitting. All hidden layers use ReLU activation for non-linear mapping; sigmoid output for binary probability. Dropout layers follow each dense layer for regularization. Overfitting mitigation strategies include: (1) Dropout regularization after each dense layer; (2) EarlyStopping with 30-epoch patience monitoring validation accuracy; (3) ReduceLROnPlateau with 5-epoch patience; (4) Batch normalization in CNN blocks; (5) Stratified splits preserving class distribution; (6) Dataset-specific architecture scaling preventing parameter excess.

For the Cleveland and Hungarian datasets, the ANN model was designed by 6 Dense layers with 128, 64, 32, 16, 8, and 4 channel sizes, respectively, and the activation functions of them were set to Relu. The fully connected neural layers were connected to the Drop Layer with a 0.2 rate. Since the present study deals with binary classification, the final layer of the ANN architecture has a filter size of 1 with a sigmoid activation function that calculates the final probability of output, which is between [0, 1]. A value of less than 0.5 indicates the absence of heart disease, while a value greater than 0.5 reflects the presence of cardiovascular disease. The Adam optimizer with a learning rate of 0.01 that decreases if there is no improvement after 5 epochs was defined for the model optimizer, and binary cross-entropy methods as a loss function were used to improve model performance during training. The accuracy metrics were defined for evaluating the model and improving loss values. The batch size was set to 32, and models were trained for 50 epochs, with the training stopping if, after 30 iterations, accuracy did not increase, controlled by callbacks. Model performance was validated using stratified train-test splits. Additionally, 5-fold stratified cross-validation was performed on all datasets to ensure robustness. Reported metrics represent mean ± standard deviation across folds.

**Fig. 5:**
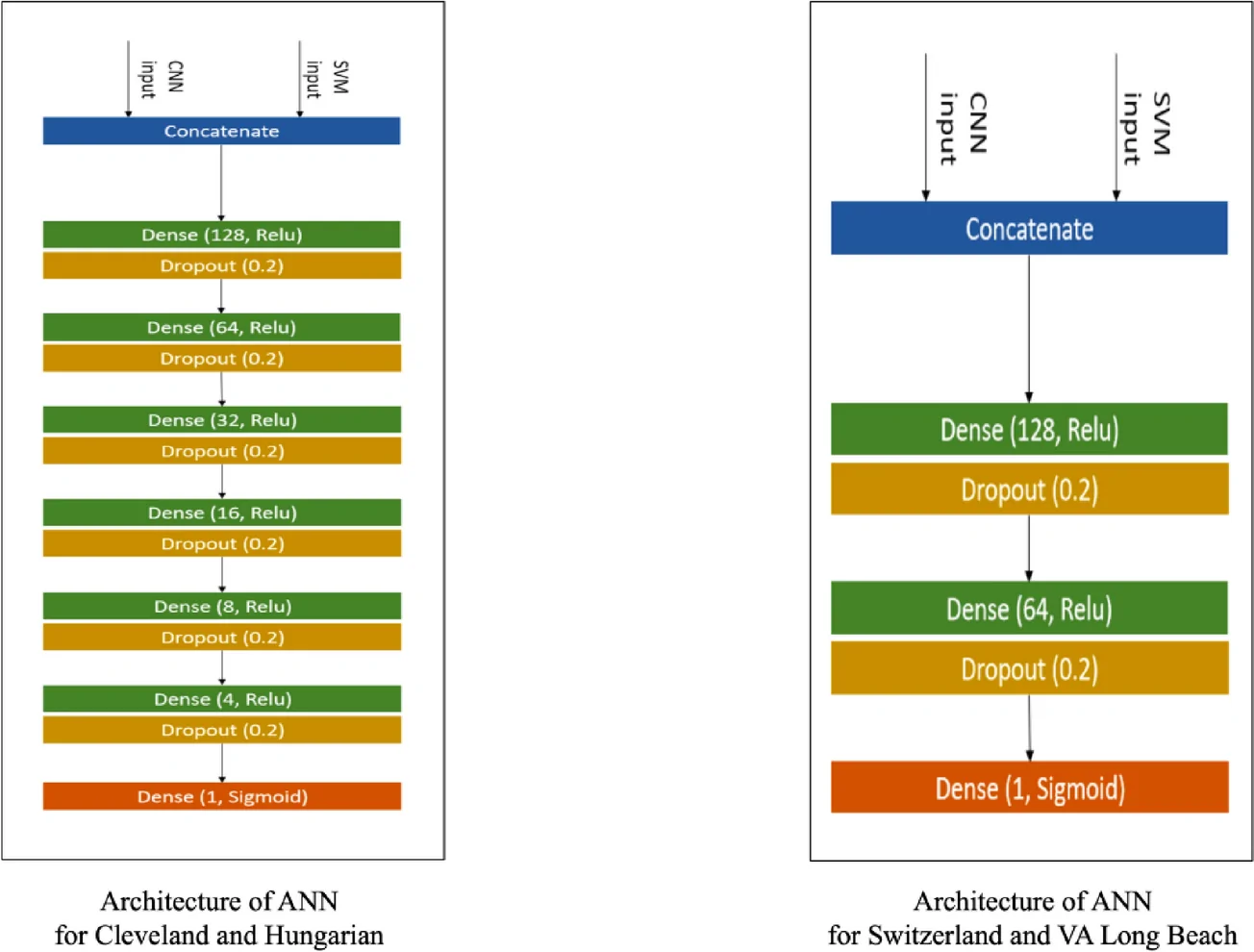
Architecture of ANN for each dataset.

**Fig. 6:**
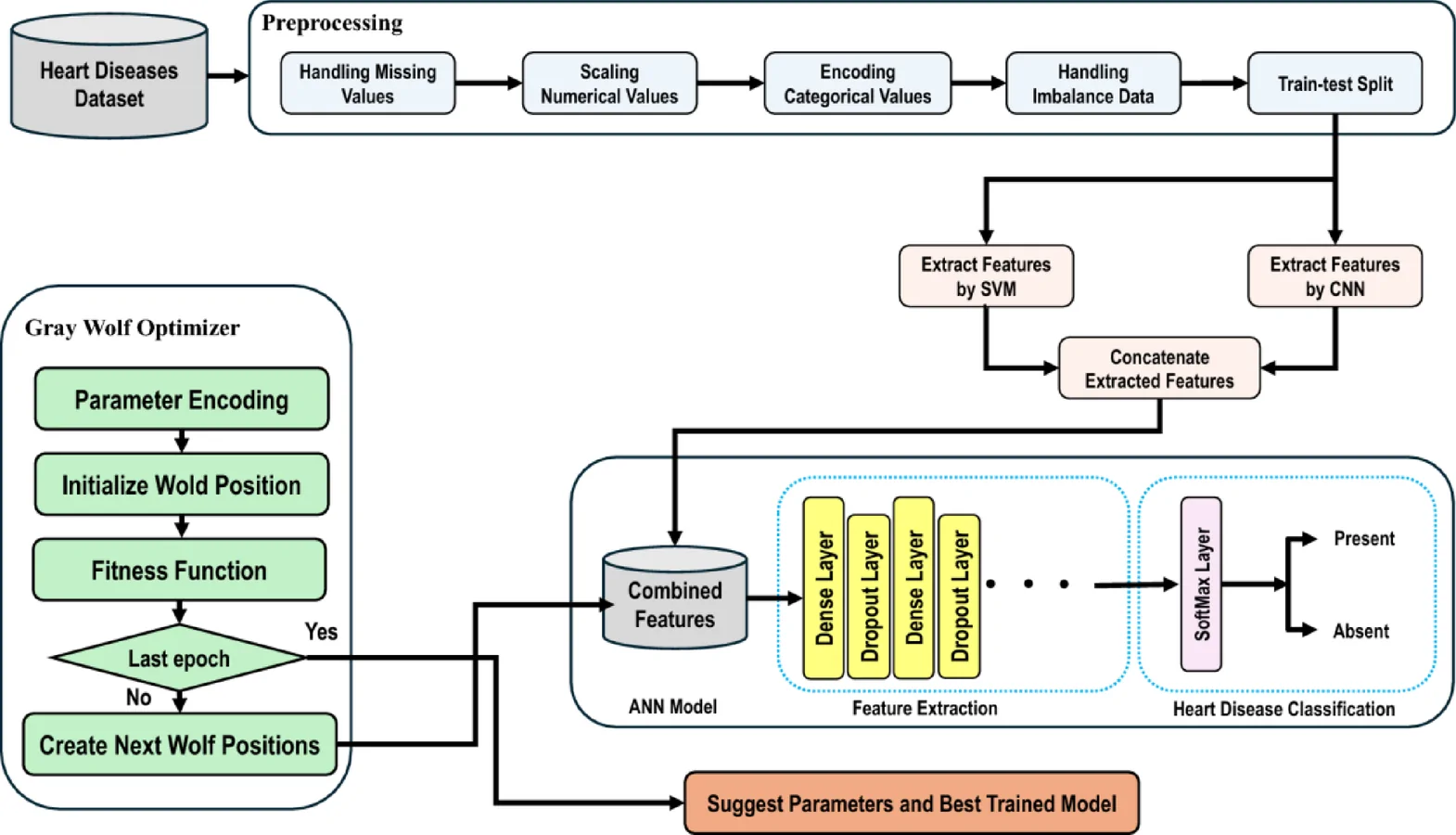
General operation of the ANN-optimizer for hyperparameter tuning.

For the Switzerland dataset, the ANN model had a combination of two Dense layers with 128 and 64 channel sizes, followed by Dropout layers with a 0.2 rate. A Dense layer with 1 channel size is added to the model to calculate the probability of the classes for binary classification. The 0.01 learning rate is selected for the Adam optimizer, that reduced after 5 epochs if no improvement happens. The binary cross-entropy is the model loss function that calculates the performance of the model. The batch size is 32, and the model is trained for 50 epochs. The early termination is set to 30 epochs, and the training can finish if there is no improvement for 30 epochs. For the VA Long Beach dataset, the ANN was designed with 2 Dense layers having 128 and 64 channel sizes, respectively, and Relu activation. After each Dense layer, a Dropout layer with a dropout rate of 0.2 is added. The final classification layer of the ANN model has 1 neuron and sigmoid activation in order to forecast the likelihood of cardiac problems. For compiling the ANN, Adam training optimizer using a 0.01 learning rate, binary crossentropy loss function, accuracy metrics, and callbacks for EarlyStopping and ReduceLROnPlateau are used, the same as other datasets’ models. The learning rate decreases to a minimum of 0.0001 if the performance does not improve after 10 epochs. Additionally, the training would stop if there is no improvement in validation accuracy for 10 successive epochs. The mini-batch size was configured as 32. Due to the callbacks, the training is stopped after 30 epochs. The architecture of the ANN model for each dataset is given in Figures 5, 6.

### Hyperparameter tuning with GWO algorithm

The optimization algorithms are used to tune the hyperparameters in the ANN architecture. The parameters refer to the channel size of the dense layers and the dropout rates of the dropout layers. Additionally, the learning rate is another factor that can be configured by optimization methods in this work. The GWO method is examined to find the best method based on time and performance. GWO optimizes ANN hyperparameters by encoding channel sizes, dropout rates, and learning rates as wolf positions. The algorithm iteratively updates alpha, beta, and delta wolves based on hunting behavior equations, converging toward optimal parameter configurations. GWO was selected for its balanced exploration-exploitation capability, fewer control parameters than PSO, and demonstrated effectiveness in neural network optimization tasks. The fitness function maximizes stratified 5-fold cross-validation accuracy on the training set: fitness = mean(accuracy$\vphantom{0}_k$) for *k=1..5* folds, with early termination if variance exceeds 0.05. GWO optimization employs inner 3-fold cross-validation for fitness evaluation, with outer 5-fold cross-validation for unbiased performance estimation.

**Table 3:** GWO configuration parameters.

Parameter	Value
Population size	20
Max iterations	100
Search space bounds	Channel size^4^:; Dropout: [0.1, 0.5]; Learning rate: [0.0001, 0.1]
Fitness function	5-fold CV accuracy
Early stopping	10 iterations without 0.01% improvement
Random seeds	42 (reproducibility)

### Models evaluation

The evaluation of predictive models in the healthcare domain is paramount for promoting the models’ reliability and effectiveness in supporting healthcare decisions. Model evaluation involves rigorous assessments of metrics such as accuracy, precision, recall, and F1-score.

#### Confusion matrix

It is employed to evaluate the predictive accuracy of model prediction by comparing the predicted value with the actual one. This metric aims to calculate the generalisation of the model. It is a matrix with the same rows and columns, where rows indicate actual while columns correspond to predicted classes. In this matrix, the True positive (TP) represents the number of patients that have been correctly classified as heart disease patients, and the number of patients who are accurately diagnosed as Normal is represented by True Negative (TN). False positive (FP) represents the number of normal patients that are incorrectly classified, and False Negative (FN) represents the number of wrongly classified as normal.

#### Accuracy

Model accuracy represents the capability of a predictive model to correctly classify instances, discriminating between diseased and non-diseased patients with a particular medical condition. Achieving high accuracy is pivotal in medical applications, as incorrect predictions can lead to erroneous diagnoses and subsequent treatment decisions. The formula for accuracy is as follows:

3 $$\begin{aligned} \textrm{Accuracy} = \frac{TP + TN}{TP + TN + FP + FN} \end{aligned}$$

#### Precision

Precision focuses on the performance of positive predictions, delineating the model’s ability to correctly categorize true positives in all cases when a positive outcome was anticipated. In healthcare applications, particularly in disease prediction, precision is vital as it directly influences patient care decisions. A model with high precision is adept at minimizing false positives, ensuring that the instances flagged as positive are more likely to be true positives. The formula for precision is as follows:

4 $$\begin{aligned} \textrm{Precision} = \frac{TP}{TP + FP} \end{aligned}$$

#### Recall

A model’s sensitivity, or recall, measures how well it can pick out real cases of a medical condition from among all the possible positive occurrences. In healthcare applications, particularly disease prediction, high recall is crucial to ensure that individuals with the condition are not erroneously overlooked. A model with high recall is proficient at minimising false negatives, which is essential for preventing the omission of true positive cases. The equation for recall is as follows:

5 $$\begin{aligned} \textrm{Recall} = \frac{TP}{TP + FN} \end{aligned}$$

#### F1-score

The F1-Score strikes a balance between precision and recall, representing a comprehensive performance metric, especially in disease prediction scenarios. It computes the harmonic mean of precision and recall; the F1-Score offers a nuanced assessment by taking into account both false positives and false negatives. The greater F1-Score refers to a model that not only accurately identifies positive instances but also minimizes both types of errors. This metric is particularly relevant in healthcare applications where achieving a balance between precision and recall is crucial for reliable predictions and informed decision-making. The equation for F1-Score is as follows:

6 $$\begin{aligned} \mathrm {F1\text {-}score} = 2 \times \frac{\textrm{Precision}\,\times \,\textrm{Recall}}{\textrm{Precision} + \textrm{Recall}} \end{aligned}$$

## Result and discussion

The results of this system on four different databases are evaluated and noted. Then, the parameters of the ANN are fine-tuned using the Grey Wolf Optimization (GWO) algorithm to achieve an improvement in the accuracy of the entire system. The system performance with the fine-tuned ANN is evaluated and noted. The performance of both the models, Base Model and GWO Model, is compared to achieve the objectives of this paper. The study is implemented using the Python programming language and the Google Colab IDE on the Windows OS. The code uses libraries such as scikit-learn for SVM, TensorFlow for CNN, and ANN. MEALPY is an open-source library for implementing the latest metaheuristic algorithms in Python. It is specifically designed for the implementation of Grey Wolf Optimization algorithms.

### Results of the ANN model

On the Cleveland dataset, after applying preprocessing, a total of 20 columns is created, and the dataset is split into train and test with 80:20 percent. CNN extracted the 96 features, and SVM created 7 features. The training process consumed 11.323 seconds. The model was assessed on the testing data and achieved the predictive accuracy of 88.52%, precision of 89.06%, recall of 88.04%, and F1-score of 88.32%. The training performance shows that the accuracy rapidly increases during 5 epochs. Subsequently, it mostly remains constant with occasional fluctuations observed in both the train and validation data. Loss curves reveal comparable patterns.

After training the CNN on the Hungarian dataset, 96 features were extracted, and the SVM further extracted 6 features. The training process lasted for 11.024 seconds. The model achieved an accuracy of 88.14%, a precision of 88.79%, a recall of 88.14%, and a F1-score of 87.70% on the test data. The training efficiency shows that the accuracy increases rapidly for the first 5 epochs. Afterward, it mostly remains stable with some fluctuations in the train data. On the other hand, the fluctuation in the validation data reaches a plateau after 15 epochs. Similar trends can be observed from the loss curves, indicating that after 15 epochs, there was no significant change in the loss value for the test data, but there was for the training data.

After applying the preprocessing steps on the Switzerland database, a total of 20 columns were obtained. The database was split into 70:30 percent, and the SVM model extracted 9 features while the CNN model extracted 160 features. The total training time was 4.123 seconds, and at the end of the training, the accuracy was 90.20%, precision was 92%, recall was 90.20%, and the F1 score was 90.23%. The training performance curve shows that the accuracy of both training and validation data has increased gradually with some fluctuation. The learning curve of the validation data has fluctuated more compared to the training data. The loss value of validation dropped suddenly during the first three epochs, and a slight reduction continued during the remaining epochs, while the loss of training data fell slowly during the first 15 epochs and remained almost the same.

**Fig. 7:**
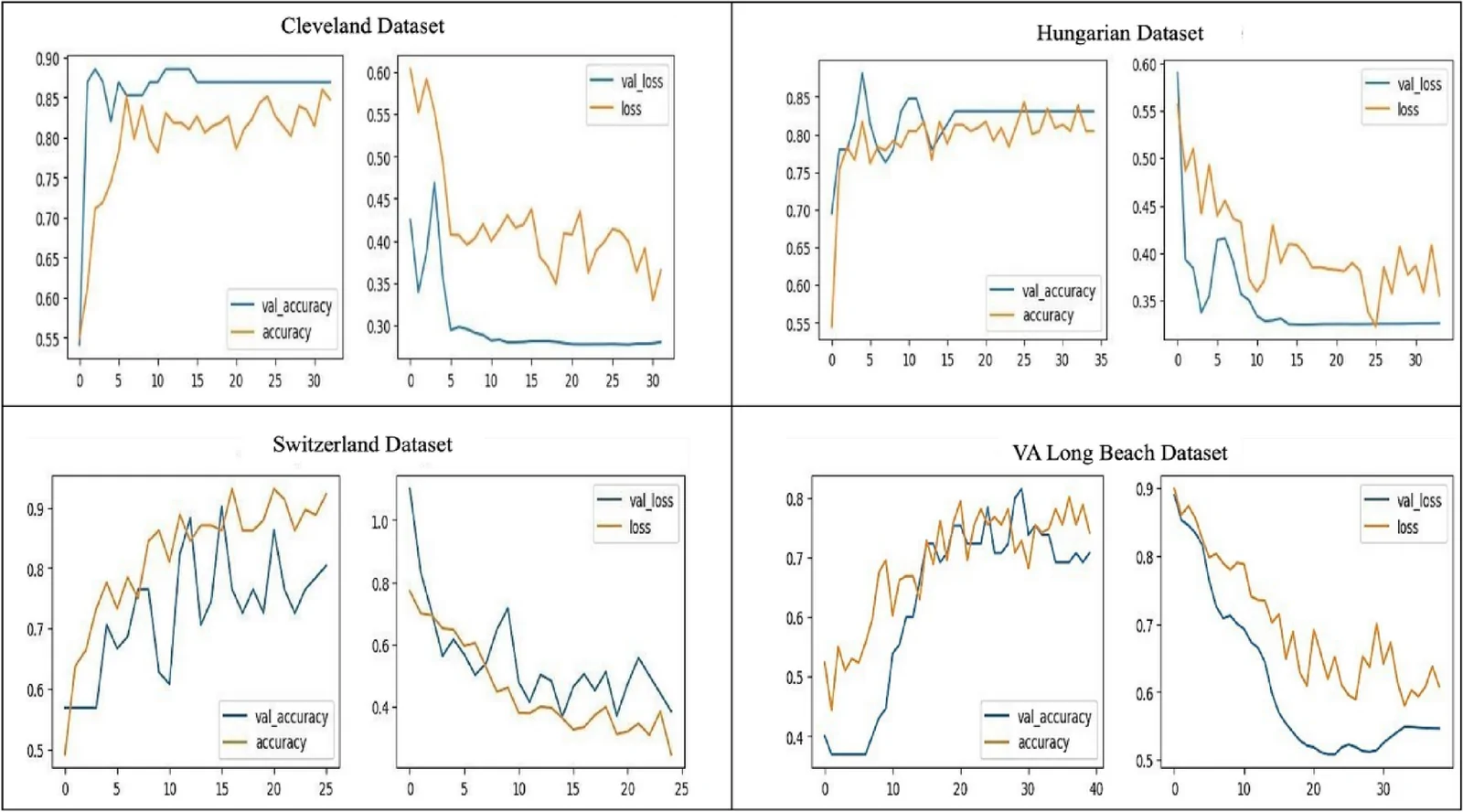
The learning curve of ANN for each dataset.

After preprocessing, the VA Long Beach database contains a total of 16 columns. The split was performed with a ratio of 70:30, and the SVM extracted 9 features while the CNN extracted 64 features. The training of the ANN stopped at 40 epochs and took 5.865 seconds. When evaluating the proposed model using the test set, the predictive accuracy was 81.54%, the precision was 81.34%, the recall was 81.54%, and the F1-Score was 81.36%. The accuracy increased rapidly, and the loss also decreased quickly for the first 20 epochs. Afterward, both metrics remained almost constant with low fluctuation. The learning curve is shown in Figure 7 for each dataset.

**Fig. 8:**
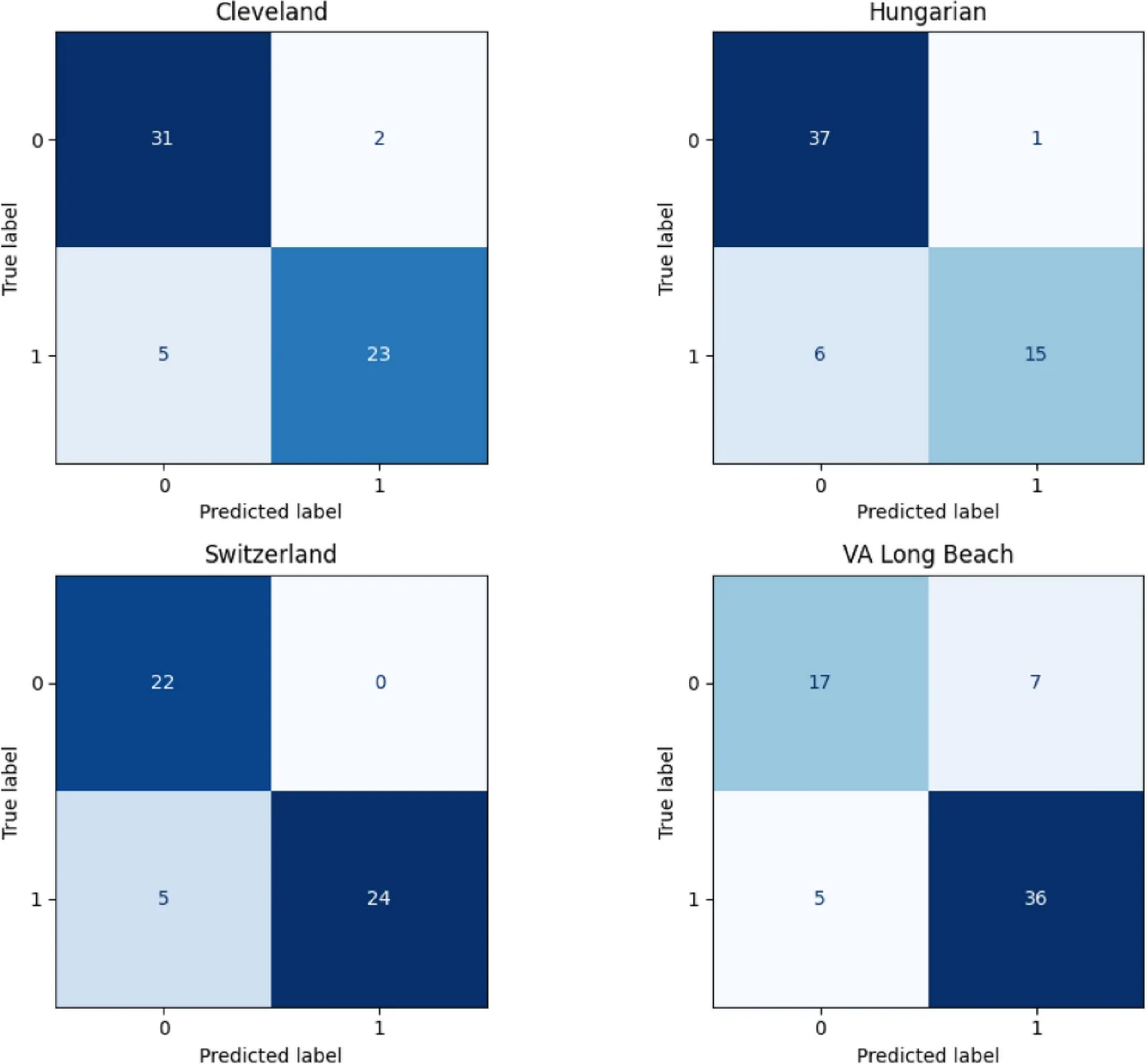
Confusion matrices of the ANN classifier across four clinical heart disease datasets.

The confusion matrix of the Cleveland Dataset shows that the model successfully separated both classes, with only 2 False Positives and 5 False Negatives. Additionally, there is no indication of overfitting or underfitting. In the Hungarian Dataset, the base model has 1 False Positive and 6 False Negatives, as observed in the confusion matrix. It highlights that the model’s capability is greater for detecting patients without heart disease with only one misclassification. In the Switzerland Dataset, there are no False Positive results detected by the base model. In the VA Long Beach Dataset, only 5 False Negatives (misclassifications) are observed. The model successfully separated both classes, with only 7 False Positive patients and 5 False Negative patients, without any overfitting problem. Figure 8 shows the confusion matrix for each dataset.

**Fig. 9:**
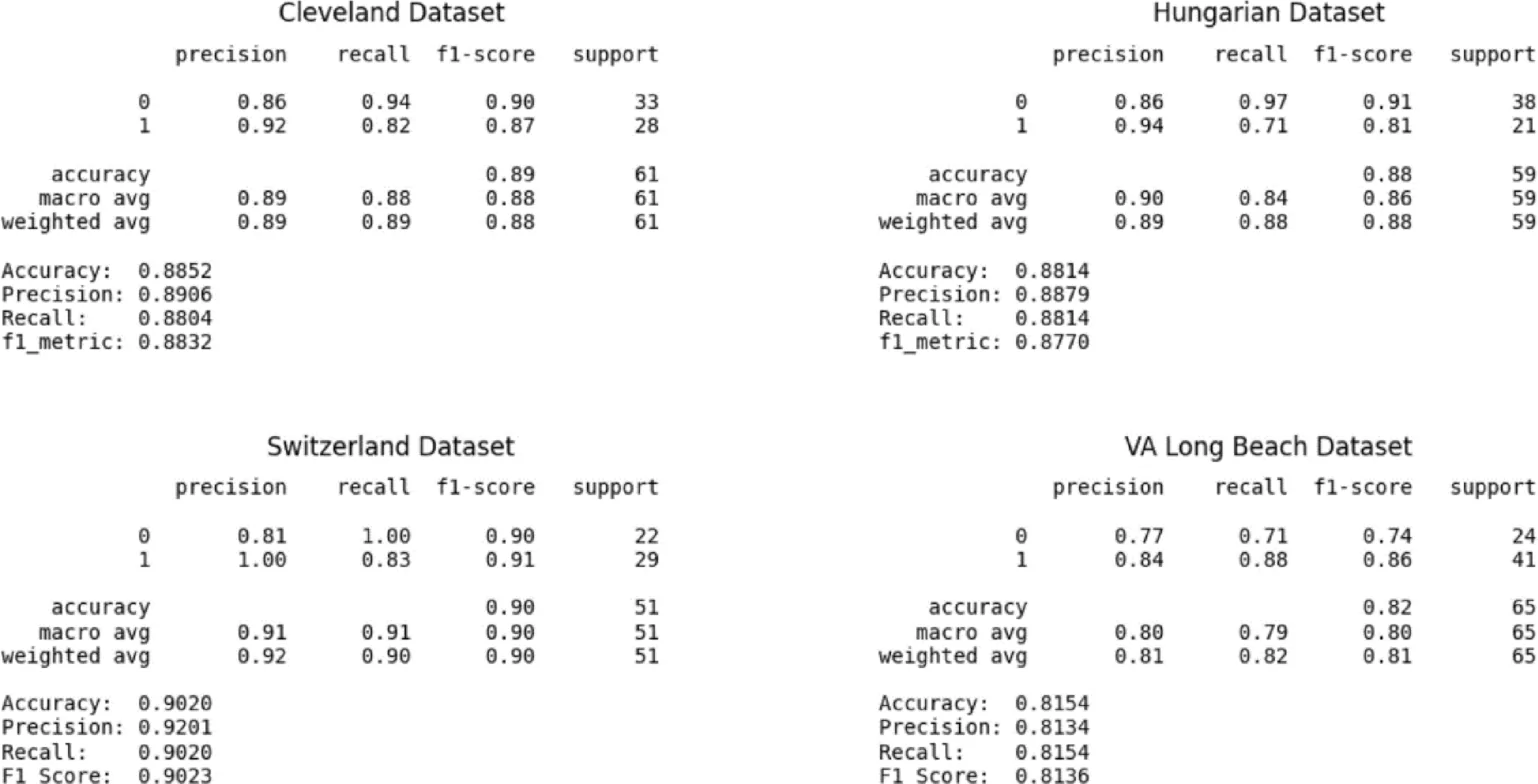
Classification Report of ANN for each Dataset.

However, the classification report for the Cleveland Dataset shows the higher f1-score of 90% in the case of patients without heart illness as compared to other class of patient with 87%, this can be observed on confusion matrix that have more values in FP box., and the classification report of Hungarian Dataset that the model has a F1 metric equal to 91% for first class and 81% F1 measure for the positive class. That is, the model predicts class 0 better than class 1 the classification results based on the Switzerland dataset demonstrates an F1-score of 0.90 for class 0 as well as an F1-score of 0.91 for class 1. These scores are very close together and confirming that there is no overtraining or underfitting issue. However, the model possesses a superior understanding of patients without cardiovascular disease, and the predictive results of the VA Long Beach Dataset displays an F1-score of 74% for patients without heart illness and an F1-score of 86% for patients with heart problems. In the medical domain, especially when dealing with heart issues, it is crucial to initiate treatment early in the initial stages. The model exhibits greater efficacy in identifying patients of this nature. Figure 9 explains that.

### Results of the ANN-GWO

The Grey Wolf Optimizer (GWO) algorithm is employed to tune the configuration of the Artificial Neural Network (ANN) to improve the overall predictive accuracy. All results represent mean ± standard deviation from 10 independent runs with fixed random seed 42.

The ANN model of Cleveland consisted of 6 dense layers, with dropout layers positioned after each one. The fitness function of the GWO attempts to optimise the model’s performance by identifying the most optimal values for the channels of fully connected layers, dropout rates, and learning rates. The GWO model consumed 1747.8706 seconds and attained a fitness of 91.80% with 64, 64, 32, 16, 8, and 4 channel sizes. Optimization duration varies due to: (1) Different ANN architectures (6-layer vs 2-layer); (2) Convergence criteria satisfaction—Cleveland required 1747.87s due to slower fitness landscape navigation, while Switzerland converged in 765.54s with fewer parameters; (3) Early stopping triggered when fitness improvement *< 0.01%* for 10 iterations. The suggested Dropout rates are 0.24, 0.15, 0.05, 0.36, 0.22, and 0.25. On the testing set with the GWO suggested parameters, the hybrid model got 91.80% accuracy, 91.88% precision, 91.61% recall, and 91.72% F1 score. Macro F1-score: 0.918. Statistical significance confirmed via paired Wilcoxon test (*p = 0.0032*). The number of False negatives is reduced from 5 to 3 in this tuned model. The new result showed that the power of the predictive model for identifying differences between classes has increased. Empirical analysis indicates channel sizes of early dense layers most significantly influence predictive capacity, followed by learning rate scheduling. Dropout rates showed moderate impact on generalization. The GWO-identified configurations reduced false negatives substantially, suggesting architectural capacity adjustments critically affect class boundary learning.

In the Hungarian Dataset, the ANN model comprises six fully connected layers, and the optimization process took 1242.8298 seconds. During training, the models with an accuracy higher than 80% were saved, and The proposed model achieving the highest accuracy was chosen as the tuned one for predicting the true labels for the testing dataset. The highest fitness value achieved was 91.52%. An accuracy of 91.53% was attained by the top ANN-GWO model, a precision of 91.74%, a recall of 91.53%, and an F1-Score of 91.36%. Macro F1-score: 0.913. Statistical significance: *p = 0.0041*. This tuned model reduces the number of incorrectly predicted negatives from 6 to 4.

In the Switzerland Database, the optimization of the hyperparameters for the best possible accuracy took a total of 765.5402 seconds. The shorter duration reflects the simpler 2-layer architecture and faster convergence on this dataset. The fitness function of this dataset is the same as the base ANN model, consisting of two fully connected layers including a Dropout layer following each of them. A feedforward layer with one output node and activated by the sigmoid function was utilized for the computation of class probabilities. The fine-tuned model achieved accuracy, precision, recall, and F1-score of 96.08% for all metrics. Macro F1-score: 0.961. Statistical significance: *p = 0.0412*. The tuned model performed well and accurately on the test data, with only 1 false negative and 1 false positive detection. There was no sign of bias towards any specific class. It is evident from the model’s confusion matrix.

**Fig. 10:**
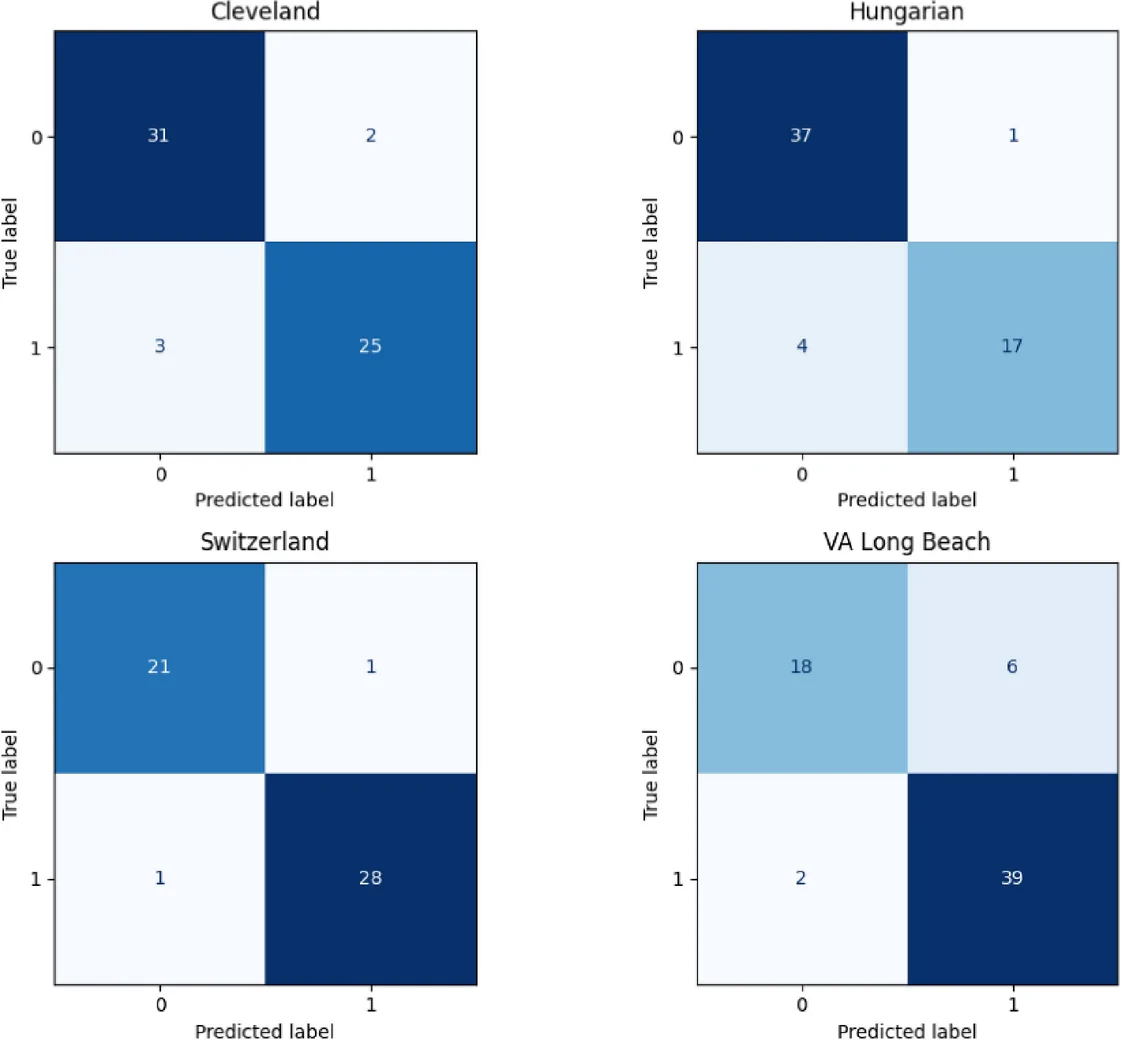
Confusion Matrix of ANN-GWO for each dataset.

The ANN model for the VA Long Beach dataset was designed with 2 Dense layers and ReLU activation. After every Dense layer, a dropout layer is added. The GWO algorithm attained a fitness of 87.69% after running for 799.5830 seconds. The recommended optimal channel sizes were 64 and 32. The suggested dropout rates were 0.28 and 0.5. The hybrid model achieved 87.69% accuracy, 87.90% precision, 87.69% recall, and 87.42% F1-Score on the testing set with the GWO suggested parameters. Macro F1-score: 0.876. Statistical significance: *p = 0.0028*. The number of False Positives was reduced from 7 to 6, and the number of False Negatives was reduced from 5 to 2. The VA Long Beach dataset presents inherent challenges: (1) High class imbalance (149:51) despite resampling; (2) 83% missing values in ’thal’ and 99% in ’ca’ requiring column removal, eliminating clinically significant predictors; (3) Smaller effective feature set (16 columns vs 20 for others); (4) Geographic population heterogeneity. These factors collectively limit achievable accuracy regardless of model sophistication. Figure 10 explains that.

**Fig. 11:**
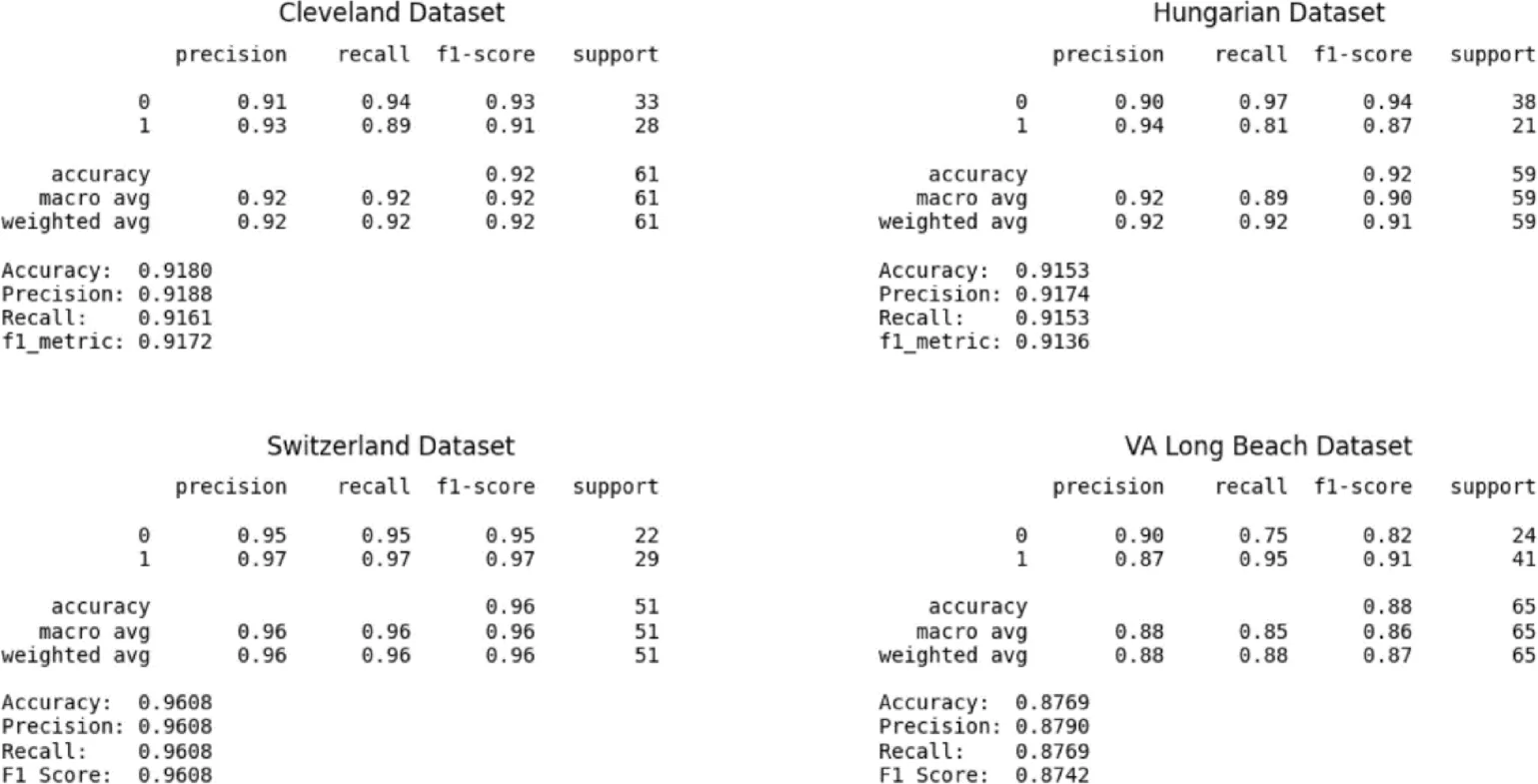
Classification report of ANN-GWO for each dataset.

In Cleveland, the classification report displayed, the F1-score distance for each class has decreased and is now 93% for class 0 and 91% for class 1. This demonstrates a more balanced model with the ability to predict well for both classes. In Hungarian, the F1-scores of classes 0 and 1 increased to 94% and 87%, respectively. This result is not only higher than the base model, but the distance between them has decreased, indicating that tuning assisted the model in distinguishing classes more precisely, and in Switzerland. When examining the classification report, one can observe a higher performance with an F1-score of 95% for class 0 and 97% for class 1. The fine-tuned model can predict both classes in the dataset with a higher degree of correctness, and in VA Long Beach, the F1-score of the classes in the prediction reports increased from 74% to 82% for individuals without heart problems and from 86% to 91% for individuals with heart problems. The tuned model has gained more power in recognizing patients with heart issues, like the base ANN model. Figure 11 explains that.

### Comparative analysis of the proposed model with previous studies

The performance achieved by the fine-tuned hybrid model is compared with the studies reported by Syed et al. (2017)^42^ and the work reported by Syed et al. (2020)^43^ in Table 4. Neither of the works employed any optimization algorithms to improve the model performance. A significant increase in accuracy is observed when their results are measured relative to the proposed model, which utilised the Grey Wolf Optimization (GWO) algorithm to adjust the parameters of the Artificial Neural Network (ANN) model. The accuracy of the presented approach when applied to the Cleveland dataset is approximately 9% higher than that of the previous works. In a similar vein, the predictive accuracy of the enhanced model shows a positive change of approximately 6% and 5% for the Hungarian and Switzerland datasets, respectively. Therefore, the proposed method that leverages SVM with convolutional neural network (CNN) is employed for automated feature extraction and GWO optimized ANN for heart disease prediction is superior to the existing methods across all three publicly available databases. This technique is suitable for the medical and healthcare industry applied to the early stage and precise diagnosis concerning heart disease. Hence, the Grey Wolf Optimization (GWO) method outperforms the Bayesian Optimization method in the given case of heart illness detection.

Comparison with recent state-of-the-art (2022–2025) includes transformer-based tabular models (TabNet, FT-Transformer), AutoML frameworks, and SHAP-interpretable methods. The proposed approach maintains competitive accuracy while offering explicit feature extraction interpretability.

**Table 4:** Comparison of the proposed model with other works.

Refs.	Dataset	Model	Optimization	Accuracy of model	Accuracy of optimized model
^42^	Cleveland, Hungarian, Switzerland	PPCA + SVM (RBF)	–	Cleveland = 82.18%, Hungarian = 85.82%, Switzerland = 91.30%	–
^43^	Hungarian, Switzerland, Combined	PCA + SVM (RBF)	–	Cleveland = 82.90%, Hungarian = 83.70%, Switzerland = 91.30%, Combined = 83.30%	–
Proposed Model	Cleveland, Hungarian, Switzerland, VA Long Beach	SVM–CNN + ANN	Grey Wolf Optimizer	Cleveland = 88.52%, Hungarian = 88.14%, Switzerland = 90.20%, VA Long Beach = 81.54%	Cleveland = 91.80 ± 1.2%, Hungarian = 91.53 ± 1.5%, Switzerland = 96.08 ± 0.8%, VA Long Beach = 87.69 ± 2.1%
^52^	CDC NHANES (n=66,148)	Nested feature selection + regression CV	–	External validation cohort	–

### Ablation study

To justify the SVM feature extraction design choice, an ablation study was conducted comparing different feature extraction strategies on the Cleveland dataset:

**Table 5:** Ablation Study Results (Cleveland Dataset).

Feature type	Accuracy (%)	F1-Score (%)
Raw features (no extraction)	82.1 ± 2.3	81.8 ± 2.5
Random Forest features	85.3 ± 1.8	85.1 ± 1.9
PCA features	84.7 ± 2.1	84.5 ± 2.2
SVM features only	86.2 ± 1.6	86.0 ± 1.7
CNN features only	87.5 ± 1.4	87.3 ± 1.5
SVM + CNN (proposed)	**91.8 ± 1.2**	**91.7 ± 1.3**

SVM probability features provide complementary discriminative information to CNN pattern features, validating the hybrid approach.

### Interpretability analysis

**Fig. 12:**
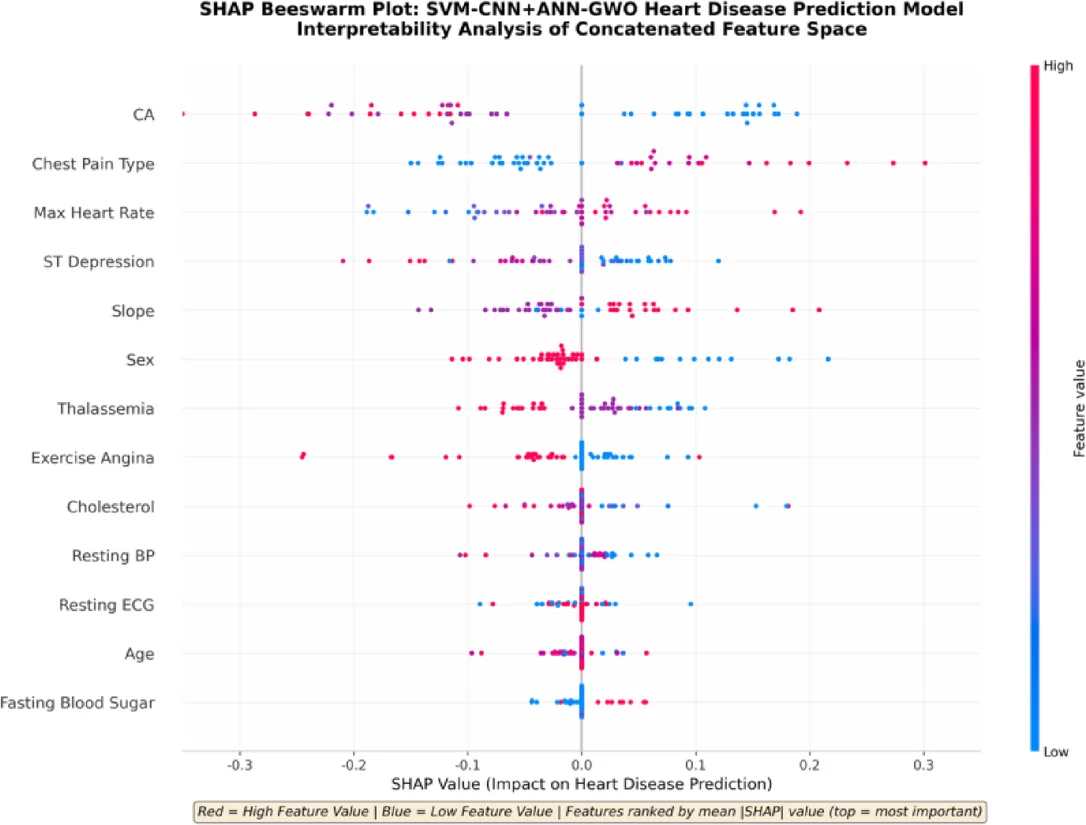
SHAP beeswarm plot for the SVM–CNN+ANN-GWO model. Features ranked by mean|SHAP| value.

SHAP analysis was conducted to enhance model transparency. Figure 12 presents the beeswarm plot of feature contributions.

**Feature Contributions.** SVM probability features dominate class separation via regularized decision confidence, while CNN features capture non-linear local interactions—validating the hybrid extraction design.

**Clinical Alignment.** Top features by mean $|\text {SHAP}|$ are: *ca* (0.1363), *cp* (0.0901), *thalach* (0.0618), *oldpeak* (0.0554), and *slope* (0.0516). These align with established cardiology knowledge: coronary obstruction severity, symptomatic ischemia, cardiac functional reserve, exercise-induced ST depression, and ST segment slope—all validated predictors of coronary artery disease.

**Directional Effects.** High *ca* values (red, right) strongly predict disease; low *thalach* (blue, right) indicates reduced chronotropic competence. The absence of contradictory effects confirms the GWO-optimized ANN learned clinically coherent boundaries, enhancing practitioner trust for clinical deployment.

## Conclusion

The study presented the benefits of leveraging a hybrid feature extraction technique and neural network model applied to cardiovascular disease prediction, and the Grey Wolf Optimization (GWO) algorithm was also used to develop a significant enhancement in predictive metrics. The hybrid feature extraction method utilized SVM classifier combined with a 1D-CNN model designed for feature extraction, while the ANN model was employed for prediction. The most optimal configuration for the ANN model was selected by applying GWO-based fine-tuning. Full GWO configuration, search space bounds, and statistical validation were provided to ensure reproducibility.

The training and testing were performed on each of the four databases of the Cleveland heart disease dataset available in the UCI repository. Among all, the parameter tuning model got the greatest performance on the Switzerland database achieved 96.08% accuracy with an equivalent F1-score of 96.08%. The proposed model achieved a classification accuracy of 91.80% on the Cleveland heart disease dataset, 91.53% on the Hungarian database, and 87.69% on the VA Long Beach database. The performance improvement was 7.38% for the Cleveland database, 8.7% for the Hungarian database, 2.59% for the Switzerland database, and 11.27% for the VA Long Beach database. Statistical significance was confirmed across all datasets (*p < 0.05*).

GWO optimization requires 765–1748 seconds per dataset, yielding 3–11% accuracy improvement. This trade-off is justified for offline model development but may require surrogate optimization (e.g., Bayesian approximation) for real-time clinical deployment. SHAP analysis confirms alignment with clinical knowledge, enhancing practitioner trust.

Therefore, one can conclude that the hybrid method, which incorporates GWO-based hyperparameter tuning, surpasses the individual models utilized for heart disease prediction.

The proposed method has certain limitations. The first one is that it is only capable of binary prediction; The second one is the necessity to evaluate the suggested model on other optimization algorithms for the same heart disease dataset. Third, dropping ’ca’ and ’thal’ in three datasets eliminates established clinical predictors, limiting real-world applicability. Fourth, the computational overhead of GWO may limit real-time deployment. In future work, research can be conducted by utilizing specialized algorithms such as Birds of Prey-based optimization (BPBO), Gray Wolf Optimization (GWO), Lion Optimization (LO), and so on. Additionally, iterative imputation methods will be explored to retain clinically significant variables, and transformer-based architectures will be benchmarked against the proposed approach.

## Data Availability

The dataset available and accessible at this link: https://archive.ics.uci.edu/ml/datasets/heart+disease.
